# An Elementary Summary of Physiology

**Published:** 1817-12

**Authors:** 


### PART II.

### COMPREHENSIVE ANALYTICAL REVIEW OF MEDICAL LITERATURE.

"Tros, tyriusve, nobis nullo discrimine agetur."

*An Elementary Summary of Physiology.* By F. MAJENDIE, Doctor of Medicine of the Faculty of Paris; Professor of Anatomy, Physiology, and Semeiology; Member of the Societè Philomatique, and the Societè Médicale d'Emulation; Associate of the Medical Society of Stockholm, &c. Translated from the French, by a Member of the Medico-Chirurgical Society.—Vol. I. containing *Preliminary Observations; the History of Sight, Hearing, Smell, Taste, Touch, Intellect, Instinct, the Passions, Voice, Attitudes, and Motions.* 8vo. pp. 211. London, 1816.

WE have often heard desponding talent complain, that there is, in this late age, no sufficient outlet for its exertions; that the fairest flowers of Parnassus have been already plucked, and the richest fruits of discovery gathered; that, in short, the favoured candidates of past ages, in right of priority, have taken possession of all the best stations in the Temple of Fame; and that even the devout and successful worshippers of our days, should they reach the envied mansion, can scarcely hope to penetrate "intra limina sanctioris Aulæ," but must be content to take up their abode amongst the outer colonnades, or, at the utmost, to rest from the anxious pursuit, and cool their animation, in the vestibule.

Whether this complaint is to the full well founded, we have not, at present, leisure to inquire; but, so far as medicine and the sister sciences are concerned, we have long been accustomed to think that *physiology* affords a most favourable opening for *original genius.* In its widest acceptation, it comprehends the history of the laws of all living bodies, whether animal or vegetable—words that need no commentary to speak the immense scope which they embrace! What has hitherto been atchieved in this extensive study, has served to shew, in a more striking manner, the infancy and imbecility of our knowledge; and perhaps it might be maintained, with little risk of exaggeration, that we have as yet scarcely got beyond the first step to wisdom—namely, the consciousness of our ignorance. What has been already accomplished, though extremely meritorious, and even, in some instances, brilliant, is, comparatively speaking, only preparatory; and doubtless a multitude of important discoveries are yet in reserve, destined to immortalize the favoured individuals whom the united kindness of Nature and Fortune shall enable to bring them to light. That the æra, however, is near at hand, in which these important discoveries will be made, we are not so sanguine as to imagine. Nay, if we may judge of the future by the past, from viewing the slow course of the stream of science, sometimes retrograde in its meanderings, and at times scarcely perceptibly progressive, we are forced to admit, that many ages must pass away ere this wished-for consummation can arrive. But that it will arrive at last, we do most firmly believe. Our principles forbid us to despair of the final fortunes of human knowledge, or to doubt that its current will one day reach the full sea of perfection (*such* perfection, we mean, as is reconcilable with the recognized *real* limits of our faculties), seeing, we have observed from the past, that it is in the ordinary course of a benevolent Providence to send down, at certain seasons, a spirit to *stir* the intellectual waters of this our world, and to communicate to them a renovating influence, suited to the wants and wishes of the lovers of wisdom; that, in short, it is part of the system of the Divine Being, at such intervals as he sees meet, to enrich and gladden humanity, by giving for its guidance a genius of the first order; a purer ray of *mind,* emanating, as it were, more immediately from his own essence; capable of acting as his Interpreter, and, in the capacity of High Priest to Nature, qualified to pass within the veil, so long thrown around her primary laws. It may perhaps be fancy, but we are persuaded that *three Newtons* succeeding each other at intervals of a century or two (supposing always no great or unlooked-for catastrophe to happen to the cause of civilization), would go nigh to bring about that *millennium* in physical science of which we are speaking. It has not been our lot to fall on that distinguished age, but we can sincerely say, that we feel our bosoms dilate with aspirations for its approach, and, in a spirit that carries us forward into futurity, we do *now* most devoutly hail its advent!

Let us not be reproached with entertaining vain expectations of the *perfectibility* of the human understanding: we know its limits, and are neither enthusiasts nor dreamers; we hate Utopian systems both in theory and practice, and are conscious to ourselves, that, in the above observations, we have been actuated by no wish to dress up a paradox, or to multiply words

> "ut placeat pueris, et ut declamatio fiat;"

but we complain that difficulties in science are apt to be abandoned too hastily, on the plea of the imperfection of our faculties. Philosophers formerly, by excess in refining, attempted too much, and their failure seems to have thrown discredit on the good cause, for we now seem to be falling into the other extreme of attempting too little. By many, in these days, to rail against all speculative pursuits, to feel a decent horror at every thing like theory, and to paint, in ludicrous colours, the folly of breaking our mental strength against the barriers which have hitherto opposed the progress of science, and which, *it is taken for granted,* our intellect cannot overleap, because it has heretofore failed to do so, are vauntingly displayed as proud proofs of a *truly* philosophic mind, and of *superior* accuracy of judgment. All this we can let pass, seeing we well know

> "Manners with fortunes,—humours change with climes,
> Tenets with books,—and principles with times:" POPE.

But what we say is, that it was not by the prevalence of such *non-chalant* notions that science has hitherto been improved. Had our forefathers suffered themselves to be subdued into sloth by the *then* apparently insuperable difficulties that stood in their way; had they, instead of attempting, age after age, to overcome them, given all up as hopeless, or scoffed at the folly of the attempt; what, we ask, would now have been the sum-total of our knowledge?" Why, a nut-shell would have been too large for it!

For our own parts, we are proud to declare, that we hold contrary principles; and it must, at least, be admitted, that our doctrine is of a more cheering and comfortable cast. We are unwilling that the cause of science should be given up without a multitude of successive trials of diligence and perseverance; that the elastic spirit, which will grasp at more than it can accomplish, should be allowed to condense and stagnate in hopeless dejection; or that it should be suffered to evaporate from a careless persuasion of the impotency of its utmost efforts; because we are quite persuaded, that while the notion of the incapacity of our faculties possesses the head, it produces that very inability which it supposes. On the contrary, "ora et labora" is the best maxim in matters of science; and since our powers owe so much of their energy to our hopes, it would, we think, be better in the actual result, that nothing should be supposed impossible to discovery, unless it is plainly proved so by the known laws of Nature. Here the test of experience, viz. the uniform failure of previously-instituted experiments, is fallacious, and proves nothing; because, as Bacon says, "new things must be attempted in new ways, since we cannot expect discoveries from a repetition of former experiments."* Upon the whole, when success

---

* "Verus experientiæ ordo primo lumen accendit, deinde per lumen iter demonstrat, incipiendo ab experientiâ ordinatâ et digestâ, et minime præposterâ aut erraticâ, atque ex eâ educendo axiomata, atque ex axiomatibus constitutis rursus *experimenta nova;* quum nec verbum divinum in rerum massam absque ordine operatum sit." *Nov. Organ* LXXXII.

Again, "At non solum copia major experimentorum quærenda est et procuranda, atque etiam alterius generis quam adhuc factum est; sed seems attainable, diligence is excited, and, in the end, often crowned with a recompence beyond even its own sanguine anticipation.

Let us trust, then, notwithstanding the present imperfect state of animal physiology, that the day is coming when men will be taught to entertain better hopes of it as a perfect science.

*Pathological physiology* embraces the explanation of the laws of the human body in its healthy and diseased state, and is merely one branch of that more general division of physical study to which the foregoing observations refer: but it is, beyond doubt, the one of far most importance to mankind; for, after all, *man* is the most proper and most interesting object of study to beings who share with each other a community of condition, and are linked together in the chain of a common nature! That this portion of knowledge is yet almost in its rude preparatory form, is a fact that cannot be concealed. We have already speculated about the perfection which, we conceive, it will at last attain; but in the mean while we should fail in our duty, if we neglected what is of more immediate consequence, namely, to point out the causes which, we conceive, have hitherto operated to retard its progress. This we shall endeavour to do as briefly as possible.

The chief of these causes we deem to be the hasty and arbitrary assumptions which have been adopted from time to time, with a view of explaining the functions which, by an abridged form of expression, we call *Life.* What, in fact, are the φυσις of Hippocrates, the *archæus* of Democritus of Abdera, the εμφατον πυρ of Plato, the *moving* and *generating principle* of Aristotle, the *vital* and *animal spirits* of the other ancients; or the *vital principle, nervous fluid, vis insita, electricity,* and *galvanism,* of the moderns, but mere technical notions, drawn not very philosophically from limited and imperfect induction, and

---

etiam methodus plane alia, et ordo et processus, continuandæ et provehendæ experientiæ, introducenda. Vaga enim experientia, et se tantum sequens, mera palpatio est, et homines potius stupefacit quam informat. At cum experientia lege certâ procedet, seriatim et continenter,—de scientiis aliquid melius sperari poterit." *Nov. Organ.* C.

We need not point out to the admiration of our readers the sterling *practical* philosophy in the above passages. May the maxims of our illustrious Chancellor soon have their due weight in the science which we are treating of in the present Article! Edit.

held up, with no little vapouring, to apologize for our absolute ignorance respecting the cause of the phænomena of living bodies. On these suppositions, gratuitously taken up, reasonings have been instituted equally gratuitous and unphilosophical: if the hypothesis would not accommodate itself to facts, these have been bent and twisted till they accommodated themselves to the hypothesis. Thus, with few exceptions, the hypothetical and synthetical methods have reigned supreme in physiology, though, almost ever since the time of Bacon, they have been justly divorced from the other branches of natural and experimental study. On such a foundation, how could the edifice be expected to stand long? Accordingly, each of the hypotheses of life above alluded to, with the physiological system erected upon it, has had its season, and its temporary reputation; but all of them have proved untenable, and, like the palace of ice reared by Imperial whim (more brilliant than substantial), have, in their turn, melted away in the spring-time of a brighter knowledge, and a purer philosophy.

We have witnessed enough to despair of ever seeing physiology take its place amongst the regular sciences, until its *form* and its *course* are completely changed. It must quit its present distressing state of imperfection; it must take on the experimental form, and be prosecuted according to the analytic course: then only will it increase, if not rapidly, at least securely; and, by and by, acquire that footing amongst the most advanced natural sciences, to which it is entitled by its latent but real capacities of improvement.

Another cause of its slow progress heretofore, is the almost total neglect, till of late years, of the light and assistance of *comparative anatomy*. It is only by studying man as an object of natural history, as one of the numerous tribes of animated beings that Nature has formed, and by studying the relations that connect him with the inferior tribes of animals, and investigating variety of function and of powers as dependent upon varieties of organization, that we can ever hope to obtain a knowledge of the primary laws of animal and organic life. On this point we are glad to avail ourselves of the support of an eminent naturalist, who, for zeal, depth, and acuteness, is surpassed by none of this, or of almost any age: "C'est meme (says *Cuvier*) principalement par l'etude approfondie de ces rapports, et par la decouverte de ceux qui nous ont echappé jusqu'à present, que la physiologie a le plus d'espoir d'etendre ses limites: ainsi doit-elle regarder l'anatomie comparée comme une des plus riches sources de son perfectionnement." *Anat. Comp.* tom. i.

As physiology and pathology must inevitably go hand in hand, the latter, as might be expected, has to the full equalled, if not surpassed the other, in absurdity. In it there has been displayed the same presumptuous, hurried and regardless adoption of some one principle to which all diseases may be traced; the same unphilosophical rashness—the same contempt for, and contortion of opposing facts. From this spirit of generalizing, axioms have been deduced to the total disregard of exceptions, just as if Nature were so very simple in her operations, that a solitary cause or two can explain the various morbid phænomena of the body under disease; or, as imperfect as man in her plans, so that she must needs admit of exceptions to her elementary laws, because human rules invariably admit of exceptions! We can tell the admirers of such pathological principles, (what, indeed, better philosophy, and a humbler mind, might have told them before) that if they have assumed any axiom as an elementary law in diseases, and find a single exception to it, that *one* exception is as conclusive against their axiom as a thousand of the same sort; and they may believe with certainty, however galling and humbling the conviction, that they have not yet reached to the depth of the *primary* law.

For our parts, we have no wish to incur the suspicion of irreverence for antiquity; but we cannot help thinking, that most of the arbitrary suppositions in pathology, from the χωλη of Hippocrates, down to the *phlogiston* of Stahl; from the *pituita*, the *dry moist* of Galen, to the *viscidity* and *lentor* of Boerhaave, and from the *fermentation* of the Arabians, down to the *error loci* of the mathematical physicians, have not only operated to prevent the growth and progress of a true and satisfactory pathogeny, (that is, a doctrine of diseases founded on the whole of the human frame, and not on the morbid appearances of single parts) but have also acted as a stumbling-block in the practice of medicine, by withdrawing mens' eyes from facts, to fix them on hypotheses.

To this faulty pathology too, we conceive, is chiefly owing that keenness of controversy, which, in every age, has torn and convulsed the exercise of our art. To what else, indeed, can we attribute the dogmatism of the *methodics* and the insanity of the *empirics*—the ill-bestowed labours of the *Greek* writers, and the worse than useless comments of their *Arabian* successors—the obstinacy of the *Galenists,* the ravings of the *chemists,* and the contemptuous vauntings of the *mathematicians?*"*

Upon the whole, then, pathological physiology seems to have been hitherto kept in its infancy by the constant proneness of its professors to kindle the torch, which was to guide them on the darksome road, at the fane of the *prevailing* philosophy, and not at the shrine of Nature; and by their neglecting to found a sure philosophy for themselves on the legitimate ground of observation, experiment, and induction.

Our limits will not permit us to enter at greater length into the causes which have operated to the prejudice of the science we are now handling. We have touched slightly on the principal ones; but, to have done the subject justice, would require a monographic essay of considerable length, instead of this hasty sketch, which our time would only permit us to take.

After these introductory observations, we now proceed to the contents of the volume before us. The celebrity of its author, and the importance of the subject, demand that we should not be sparing of the space we allot to it. In such a variety of matter, however, as it comprises, a *critical* analysis of every part can hardly be expected from us: we shall, therefore, give merely a full outline of its contents; and at the end, enter into a general estimate of its merits, in which our readers will be enabled to go along with us.

The *Preface* is short, but well written; and contains some judicious observations on the most advantageous mode of cultivating science. We are happy to perceive that Professor Majendie (unlike many of his countrymen), pays a compliment to our immortal Bacon, and recognizes the influence which his writings have had in breaking the fetters of the Aristotelian philosophy, and introducing a fortunate change into all the natural sciences—into all, save physiology! What, however, more particularly pleased us in the Preface, and raised our expectation, is the following paragraphs:

* Dr. Pitcairn, one of the ablest and acutest of these *Iatro-mathematici,* concludes his work in the following *modest* terms: "Non dubito me solvisse nobile problema, quod est,—Dato morbo, invenire remedium.—Jamque opus exegi!"

"There will, therefore, above all, be found in this work facts which I have directly verified, as much as was in my power, either by observation upon the healthy or diseased subject, or by experiments upon living animals. Among these facts will be found many which are new."

"I have not, however, neglected the possible and useful application of physics, of mechanics, chemistry, &c. to the phænomena of life; perhaps they will be different from those which have been hitherto offered, for I have neglected nothing to ascertain their exactness." P. ix.

This is as it should be, and just as we would have it.

The work sets out with some preliminary observations on bodies, and their division into *ponderable* and *imponderable, simple* and *compound,* into *organic* or living, and "*inorganic, inert, brute,*" *or dead.* This is very well, but we must beg leave to quarrel with the term *inert*: it is a faulty one; and as such, we wish to see it banished from philosophical writings. Inorganized or dead matter is *not* inert, as the phænomena of chemical action, and cohesive attraction sufficiently prove. The word was a very famous one amongst the schoolmen; and, probably on that account, has continued to find favour in the sight of the moderns. We wonder, however, that it should have been employed by one of our author's accuracy and acuteness.

It is not with inorganic but with living bodies that physiology has to do: therefore, we shall pass over the lengthened tabular view of the differences between the dead and living kingdoms of Nature, which our author has given. They are different in their *form,* their *composition,* and the *laws* by which they are governed. Living bodies again are divided into *animals* and *vegetables,* and the following table we extract as succinctly pointing out the characteristic differences between them.

| " *Vegetables.* | " *Animals.* |
|---|---|
| Fixed to the soil. | Move on the surface of the soil. |
| Have carbon for the principal base of their composition. | Have azote for the base of their composition. |
| Compounded of four or five elements. | Often compounded of eight or ten elements. |
| Find and take their nourishment ready prepared around them." | Have to act upon their nourishment to render it fit for its purposes." Page 4th. |

The vegetable kingdom is dismissed by the author in like manner as the inorganic: *animal* physiology being the object of his work, he conceived it necessary, merely for the sake of clearness, to allude to the former, in his preliminary observations about the general division of bodies.

With regard to the ultimate and proximate principles of animal substances, our author has advanced nothing new. We shall not transcribe them, inasmuch as they are to be found in every elementary work on animal chemistry. The following remark on adipocere, however, is of some importance, because we know it is a received opinion, that at least one species of biliary calculus is quite analogous, in its composition, to this curious substance.

"Adipocere is found in bodies long buried in the earth; it is composed of margarine, of acid fluid fat, of an orange colouring principle, and of an odorous principle. This must not be confounded with the spermaceti of the whale, and with the biliary calculus; which, indeed, are very different from each other. M. Chevreul has proved that it does not contain a single principle analogous to them." Page 5.

The next section treats of the *organic solids* of the body. We presume all our readers know (what to persons unacquainted with anatomy would seem a paradox), that the solids constitute only *one tenth* of the weight of the whole body. This is proved by the Egyptian mummies, and by the exhumation of bodies that have been long buried in dry soils, or in the burning sands of Arabia. Professor Chaussier also has proved it directly, by putting into an oven a body weighing one hundred and twenty pounds: after several days, it became reduced to only twelve pounds.

We solicit our readers' closest attention to the following extract, and to the remarks which we feel called upon to offer in contravention of it.

"The ancients thought that all the organic solids might be ultimately reduced to a simple fibre, which they supposed to be formed of earth, oil, and iron. Haller, who admitted this idea of the ancients, allows that this fibre is visible to the eyes of the mind only; 'Invisibilis est ea fibra, solâ mentis acie distinguimus.' He might as well have said that it has no existence, as every one at present is convinced.

"The ancients besides admitted secondary fibres, which they supposed to be formed by particular modifications of the simple fibre. Hence the *nervous* fibre, the *muscular*, *parenchymatous*, and *osseous*.

"M. Professor Chaussier has, in modern times, proposed to admit four kinds of fibres, which he terms *laminary*, *nerval*, *muscular*, and *albugineous*.

"Science was nearly in this state when M. Pinel conceived the happy idea of distinguishing the organic solids, not by fibres, but by tissues or systems. Upon this division he founded many orders of diseases, but particularly the phlegmasiæ. Bichât seized this fine conception, and applied it to all the solids of animal bodies; the work which he wrote on this subject (*Traité de l'Anatomie Générale*), is his highest title to glory. M. Dupuytren has perfected Bichât's classification; M. Richerand has also pointed out several of its defects. This is the classification of tissues, rectified according to the views of MM. Dupuytren and Richerand.

Systems

- "1. Cellular

- 2. Vascular - - - - - - - - -

  - Arterial

  - Venous

  - Lymphatic

- 3. Nervous - - - - - - - - -

  - Cerebral

  - Gangliar

- 4. Osseous

- 5. Fibrous - - - - - - - - -

  - Fibrous

  - Fibro-cartilaginous

  - Dermoid

- 6. Muscular - - - - - - - - - -

  - Voluntary

  - Involuntary

- 7. Erectile

- 8. Mucous

- 9. Serous

- 10. Horny, or epidermous -

  - Pilous

  - Epidermoid

- 11. Parenchymatous - - - -

  - Glandular."

"These systems united together, and with the fluids, compose the organs or the instruments of life." Page 7th.

Here follows the Translator's note.

"The French are wrong in attributing, as they always do, the originality of this classification to Pinel. The Translator begs permission to quote a note on this subject, from an Inaugural Dissertation published at Edinburgh, by his friend Dr. Elliotson, in the year 1810.—"Ad hunc modum primus de inflammatione scripsit Carmichael Smyth, M. D. in dissertatione pulcherrima, quam Societati cuidam Londinensi, A. D. 1788, recitavit. *Medical Communications*, vol. ii.—Decem post annis elapsis, eandem divisionem secutus est Pinel. *Nosographie Philosophique*, tom. i.—Bichât postea indicavit omnes morbos hac eadem ratione utilissime considerari potuisse. *Anatomie Generale*, passim." P. 7th.

For this note he has our best thanks: we did not, indeed, know of such a passage in Dr. Elliotson's Thesis, or of Dr. Elliotson's Thesis at all; (for which want of information the very limited publicity of those productions must form our excuse*) but we did know that Dr. Carmichael Smyth, in his excellent Essay on "the Nosology of Inflammations," read before the Medical Society of London in 1788, and published two years afterwards, completely and indisputably anticipated both Pinel and Bichât in this important discovery! Nay, many years before this period, Mr. John Hunter, with his usual unrivalled acuteness in surgical pathology, had, in his public lectures, inculcated the principle that inflammation is modified by the structure of the part it affects: and though his opinions were not published in form till 1794, still manuscript extracts and abridgments of his lectures were previously in the possession of pathologists and students, and disseminated over Europe.

Thus, then, we deem it proved beyond controversy, that Mr. Hunter and Dr. C. Smyth are the rightful owners of this discovery, about which the French have made such a racket, and which they have so unjustly attempted to appropriate to themselves. It is now high time that restitution should be made, and that "execution should be done on *Cawdor*"—that is, on Pinel, who was the first, in this case, to stretch out the furtive hand and seize on the laurels of others. He deserves no quarter at our hands, for whom, of all his predecessors or contemporaries, has the self-sufficient author of the *Nosographie Philosophique* spared?

Bichât, though not a principal, was a "particeps criminis;" but his high talents, and the valuable labours he has bequeathed to posterity, so far redeem him, as to ren-

---

* This limited publicity we have often lamented as not only an actual loss to the stock of modern medical knowledge, but detrimental to that emulative spirit for excellence which ought to be cherished, and not repressed, in the candidates for medical honours. Although, we fear, many of these Theses can only claim the character of mere *Academic Exercises*, which may well be let die as soon as they have served their turn; still there are not a few, the offspring of practical knowledge, or the result of ingenious physiological experiment, which are well deserving of a better fate. Yet "all things happen alike to all," and there is one event for the talented and diligent, and for the ungifted and indolent candidate. "Omnibus una manet nox!"—Their labours are alike consigned to pseudo-immortality—to an existence silent as oblivion itself, in the unexplored recesses of the library of "Alma Mater."—*Perhaps* it may be truly said,

> "Full many a gem of purest ray serene,
> The dark and dusty shelves of *Alma* bear."

We are inclined to think that a judicious selection and condensation of the best of these Dissertations, now and then, would be no unacceptable article to the readers of our Journal.—Edit.

der us unwilling to speak of his offence as, in this instance it merits. Besides, he is now no more; the sun of his fame has set in "the narrow house," and far be from us any unfeeling wish to agitate the hallowed stillness of the grave!"—

"—— For English justice wars not with the dead."

We are particularly surprised, not to say displeased, that such a writer as Dr. Majendie should have sanctioned the culpable and bare-faced plagiarism of M. Pinel, by complimenting him, with all the suavity of complaisance, on *his* "happy idea!" We suspect, however, that notwithstanding the self-esteem so apparent in the literary character of the latter, he must receive this and similar panegyrics with the *gauche* and embarrassed air of the man, who, in a moment of purely *accidental forgetfulness* of the difference between *tuum* and *suum*, had helped himself to another's shirt, and was afterwards complimented on the beauty and fineness of his linen!

As if our learned author had not gone already too far, he is pleased to call in M. M. Dupuytren and Richerand to share with M. Pinel the merit of the discovery, from an opinion, probably, that its present illegal possession might be made good by the superior number of the occupants!—But, "Apollo gaudet numeris imparibus," and perhaps our ingenious author meant more than meets the eye in this, namely, some latent *calculation:* wishing to fix the discovery irrevocably amongst his countrymen, and to make the appropriation *square* with *law*, since it could not be made to square with justice, he ingeniously consigns it over to no less than 3 : that mystical figure, in this case representing the *square-root* of 9—the precise number of legal points which possession is understood to carry in its favour!—for, we suppose, it is a maxim in France, as well as amonst ourselves, that "Possession is *nine points* of the law!"

We know, indeed, that it is most potently believed in France, that no brain but a Frenchman's ever throbbed with a "fine conception!" This is as *clear* to most understandings *there*, as any axiom in Euclid. *Je* and *moi* seem to be the Gog and Magog of a Frenchman's idolatry; hence the constant canting about *esprits-forts* and *grandes penseès* as indigenous to the favoured soil of "the great nation." But, that Professor Majendie should not have been superior to such narrow nationality, we own, astonishes us! We have turned this subject over in our minds, and have honestly and anxiously sought for any possible way of explaining away his conduct, or of assigning it to motives other than culpable; but without success. Professors Pinel and Majendie, as well as Richerand and Dupuytren, are men of too much erudition for it to be supposed that they were ignorant of Mr. John Hunter's opinions; or, above all, that they were unacquainted with Dr. Smyth's Essay, published in a collection of so great celebrity, and commented upon in all the widely-circulated periodical works of the time. The conclusion, therefore, is, we fear, inevitable. Their offence was wilful: they are conscious of their injustice, but wish to *face it out,* and keep each other in countenance by the reciprocal cackling of compliment.

We are ever ready to acknowledge, honour, and admire the zeal, industry, acuteness, and substantial merit of the French, not merely in the various branches of our profession, but in every department of natural science; but we are not the less ready to oppose that excess of vanity and ambition (which in science, as in politics, is the besetting sin of their nation, that prompts them to compromise the claims, and encroach upon the property of their neighbours. The discoveries of our men of science are a species of proud inheritance, bequeathed to us in a manner at once solemn and memorable: they have become a valuable portion of the national property; sacred; unalienable; and associated with the most touching recollections! It is incumbent upon us to defend them with patriotic spirit. We have just emerged from a protracted, but successful, struggle against the political aggressions and encroachments of our rival-neighbours; let us now protect our scientific common-weal against a similar grasping, unjust, and predatory spirit. They have not succeeded by the sword; neither, *we* trust, shall they succeed by the pen!

Our readers may wonder at our great keenness in this matter; but, we apprehend, they will cease to do so when we tell them, that we consider the discovery in qnestion, both theoretically and practically, a very beautiful one, and a most important step gained in modern correct pathology. It opens up a view of diseases, deduced from observations of similar structure throughout the whole body, and not from the morbid changes of isolated parts; and suggests a more accurate, natural, and philosophical mode of arranging and considering these diseases. It shews how far inflammation, and even organic lesions, are modified by the structure, function, and situation of the part affected, and introduces a simplicity and uniformity of plan into the pathology, particularly of surgical diseases, highly to be appreciated. It points out also a division of the symptoms into those which are primary, general, and characteristic of all the diseases of parts of a similar structure, and those which are only occasional, accessory, or owing to the function or situation of the part implicated: For the great difference in the symptoms, which is so apt to puzzle the practitioner at the bed-side, is more owing to a difference in the structure and function of the part affected, than to any specific difference in the nature of any given disease, which, in most cases, is nearly of the same kind, and requires the same general treatment. Thus, for instance, inflammation of the cutaneous, the parenchymatous, or the cellular texture; of serous, mucous, fibrous and synovial membranes, will exhibit one primary disease under six or seven generic modifications; a circumstance of great consequence to be known in the nosological arrangement, the diagnosis, and the successful treatment of this affection, when it occupies parts either external or internal.

Our author's next Section treats of "the fluids or humours;" he divides them thus:

"1. The *blood*. 2. The *lymph*. 3. The *perspiratory fluids*, which comprehend the fluids of the cutaneous transpiration, and of that of the mucous, serous and synovial membranes, of the cellular membrane, of the adipose cells, the medullary membranes, the interior of the thyroid, the thymus, &c. 4. The *follicular fluids:* the fatty fluid of the skin, the cerumen, the secretion of the meibomeian glands, the mucus of mucous glands and follicles, of the tonsils, the glands of the cardia, the neighbourhood of the anus, the prostate, &c. 5. The *glandular fluids;* the tears, saliva, pancreatic fluid, bile, urine, the fluid of Cowper's glands, semen, milk, the fluid of the supra-renal capsules, that of the testicles and breasts of new-born infants. 6. The *chyme* and *chyle.*" Page 9.

This enumeration is sufficiently minute. Great importance has been attached to classing the fluids methodically: every classification is, in a great measure, arbitrary; but, upon the whole, we think the present one which Professor M. has adopted, to be the best, and least liable to exceptions; as it considers neither the nature of the fluids, nor their uses, or chemical qualities; but is founded upon the mode of their formation, which is the only invariable character they present. We believe M.

Chaussier is the original proposer of this mode of classification.

There is a Section of two pages about the "vital properties," but this portion of the volume we have in vain endeavoured to comprehend. The author here is brief even to darkness, and seems to forget that the first end of writing is to be understood. Since we are told that the work is intended for medical *students*, we think it was incumbent upon the translator to have added an explanatory note here, unless indeed he was in the same dilemma with ourselves, and at a loss for his author's meaning.

The following paragraph opens up our author's elementary views of the phænomena of living bodies, and is besides a fair specimen of his style and tone of composition.

"Whatever may be the number or diversity of the phænomena of the living man, it is easily seen that they may be ultimately reduced to two principal ones, *nutrition* and *vital action*; a few words upon each of these phænomena are indispensible for comprehending what will follow.

"The life of man and other organized bodies is founded upon their habitually assimilating a certain quantity of matter called *aliment*. The privation of this matter for a very limited period, necessarily causes the cessation of life. On the other hand, daily observation shews that the organs of man, and other living beings, lose every moment a certain quantity of the matter which composes them; and it is in fact from the necessity of repairing these habitual losses, that the necessity for nourishment arises. From these two data, and some others which we shall make known in our progress, it has been concluded with reason, that living bodies are not composed of the same matter at every period of their existence: it has been even said, that bodies undergo a total renovation. The ancients maintained, that this renovation takes place every seven years. Without admitting this conjecture, we shall say, that it is extremely probable, that every part of the human body experiences an intestine movement, the object of which is both to expel the particles which are no longer destined to remain in the composition of the organs, and to replace them by fresh particles. This intimate movement constitutes nutrition. It is not sensible, but its effects are; and to doubt its occurrence, would be the height of scepticism. This movement is inexplicable, and can be referred, in the present state of physiology, only to the molecular movements of chemical affinity. To say that it depends on organic sensibility and insensible organic contractility, or simply on vital force, is merely expressing the same fact in different terms. However this may be, the organs of the human body preserve and change their physical properties in virtue of their nutritive movements, or of nutrition. As our different organs present different physical properties, the nutritive movement must vary in each of them. Independently of the physical properties which every part of the body presents, there are many which offer either continually, or at more or less distant intervals, a phænomenon termed *vital action*. For example, the liver continually forms a fluid called *bile*, in virtue of a power peculiar to it; the same may be said of the kidney with respect to the urine. The voluntary muscles, under certain conditions, harden and change their form; in a word, contract. This also is an instance of a vital action. These vital actions are very important to the life of man and animals, and particularly demand the attention of the physiologist.

"Vital action depends evidently upon nutrition, and nutrition again is influenced by vital action. Thus an organ which ceases to be nourished, loses at the same time the faculty of acting; thus organs whose action is the most frequently repeated, have a more active nutrition: on the contrary, those which act less, have evidently a sluggish nutritive movement.

"The mechanism of vital action is unknown. There occurs n the organ which acts an insensible molecular movement, as inexplicable as the nutritive movement. No vital action, however simple, is an exception in this respect.

"All the phænomena of life may be thus ultimately referred to nutrition and vital action; but the molecular movements which constitute these two phænomena, are not sensible, and we must not direct our attention to them; we can study their last results only, i. e. the physical properties of organs, and the sensible effects of the vital actions; and examine how they both concur to general life. This, in fact, is the object of physiology." p. 15.

In no point in physiology has there been greater diversity than in the classification of the functions. They were formerly divided into the *vital*, the *natural*, and the *animal*; an arrangement for all practical puposes, perfectly unexceptionable. More recently, the old classification of Aristotle has been modified and revived; and the functions have been divided into those of *organic* or *automatic*; and into those of *animal* life. The first division comprehends nutrition, secretion, and, in short, all the properties that animals have in common with the vegetable kingdom; the latter class comprehends all the functions *peculiar* to animals, such as voluntary motion, sensation, intellect, and passion. This division, we esteem the most simple, and the most philosophical in its principle. Our author, however, in the work now before us, divides the functions into those which are destined to place the individual in relation with surrounding objects; those whose object is nutrition, and those which are intended for the reproduction of the species. He calls the first, "*functions* of relation;" the second "*nutritive functions;*" and the third "*generative functions.*" The first class of functions only is treated of in the present publication, the other two classes our author intends to form the subject of a subsequent volume.

(To be concluded in our next.)

---

*Medico-Chirurgical Transactions, Vol. VIII. Part I. Octavo, pp.* 315, *with Plates,* 1817.

